# Effects of the Ecdysone Receptor on the Regulation of Reproduction in *Coccinella septempunctata*

**DOI:** 10.3390/insects16060643

**Published:** 2025-06-19

**Authors:** Ying Cheng, Yuhang Zhou, Cao Li

**Affiliations:** 1 Guizhou Institute of Plant Protection, Guiyang 550006, China; 2 Guizhou Key Laboratory of Agricultural Biosecurity, Guiyang 550006, China; 3 Guizhou Branch of State Key Laboratory for Biology of Plant Diseases and Insect Pests, Guiyang 550006, China; 4 Guizhou Provincial Laboratory of Green Technology and Application Engineering of Plant Protection, Guiyang 550006, China

**Keywords:** *Coccinella septempunctata*, molting hormone, ecdysone receptor, RNAi, reproductive regulation

## Abstract

The effects of the gene encoding the ecdysone receptor (*EcR*) on the reproduction of the ladybug *Coccinella septempunctata* was evaluated. *EcR* transcription was measured by quantitative real-time PCR in ladybug adults reared on artificial diets with and without 20-hydroxyecdysone (20E). *EcR* expression levels in 5 d old male and female ladybugs supplied with the 20E-amended artificial diet were lower than expression levels in ladybugs reared on an artificial diet lacking 20E. However, *EcR* expression levels in 10 d old ladybugs supplied with the 20E artificial diet were higher than those lacking 20E supplementation. The regulatory effects of *EcR* were studied in female and male ladybugs by RNA interference. *EcR* expression in female ladybugs injected with *EcR*-dsRNA was significantly downregulated after 5 d but remained unaffected in 10 d old females. *EcR* expression levels in males microinjected with *EcR*-dsRNA were significantly lower at 5 and 10 d after microinjection than *GFP*-dsRNA-treated males. The ovary volume in females injected with *EcR*-dsRNA at 5 d was smaller than females microinjected with *GFP*-dsRNA, but volumes at 10 d were larger than in *GFP*-dsRNA-treated females. The testes of males injected with *EcR*-dsRNA were larger than those injected with *GFP*-dsRNA at 5 d but the testes at 10 d after injection with *EcR*-dsRNA were smaller than those injected with *GFP*-dsRNA. When females were microinjected with *EcR*-dsRNA and mated with noninjected males, egg production decreased by 34.80% for 20 days. When males were microinjected with *EcR*-dsRNA and mated with noninjected females, egg production decreased by 30.38% for 20 days. Injection of female and male ladybugs with *EcR*-dsRNA had no significant effect on egg hatching rates. Our results show that *EcR* plays an important role in the development of reproductive organs and egg development in *C. septempunctata*.

## Simple Summary

The ladybug *Coccinella septempunctata* L. (Coleoptera: Coccinellidae), a natural enemy, is widely used in the biological control of insect pests. At present, the primary method for producing *C. septempunctata* is to rear aphids as their diet. This method requires considerable space and is labor-intensive. To solve these problems and achieve large-scale production of ladybugs, the development of an artificial diet is needed. Previous studies have shown that adding the molting hormone (MH) to artificial diets caused a significant increase in the reproductive capacity of ladybugs. The main function of the MH is to promote physiological processes such as the tanning and hardening of the epidermis and regulate insect behavior, diapause, and reproduction. The MH regulates the transcription of downstream genes by binding to the *EcR*/*USP* complex, thereby regulating the biological response of developmental stages or tissues to hormonal signals. The impact of *EcR* on ovary and testis development and reproductive capacity in *C. septempunctata* remains unclear. In this study, we use RNAi to confirm the regulatory function of *EcR* in *C. septempunctata* reproduction. The findings enrich our understanding of MH regulation during ladybug reproduction. This study contributes to improved methods for cultivating natural enemies and will ultimately benefit pest control.

## 1. Introduction

The ladybug *Coccinella septempunctata* L. (Coleoptera: Coccinellidae) is widely distributed in Asia, Europe, and northern Africa [1]. This natural enemy is widely used in the biological control of insect pests [2,3] and possesses a large appetite, robust reproductive ability, and strong adaptability. At present, the primary method for producing *C. septempunctata* is to rear aphids on host plants and allow ladybugs to reproduce by feeding on the aphids. This method requires considerable space and is labor-intensive. To solve these problems and achieve large-scale production of ladybugs, the development of an artificial diet is needed [4]. Although efforts to formulate an artificial diet for ladybugs began in the 1950s [5], a suitable artificial diet for large-scale rearing is lacking. Problems associated with artificial diets include prolonged larval stages, low survival rates, reduced egg production, and low egg hatching rates. The emergence of these problems is sometimes related to feeding methods and environmental conditions but is primarily due to the nutritional composition of diets and hormone regulation.

The molting hormone (MH), also known as ecdysterol, is produced by the prothoracic gland. The main function of the MH is to promote physiological processes such as the tanning and hardening of the epidermis and regulate insect behavior, diapause, and reproduction [6,7,8]. In insects, the MH is primarily present as 20-hydroxyecdysone (20E). After insects ingest cholesterol or other steroid hormones from food, ecdysteroid precursors are converted to active 20E by cytochrome monooxygenases in the P450 family [9]. An early target of 20E is the ecdysone receptor (EcR)/superspiracle (USP) complex, which belongs to the nuclear receptor superfamily. The MH regulates the transcription of downstream genes (e.g., *E75*, *E78*, *FTZ-F1*, *HR4*, *HR38*, *HR39*) by binding to the EcR/USP complex, thereby regulating the biological response of developmental stages or tissues to hormonal signals [6,10,11].

In adult insects, the MH is synthesized in gonads, where it promotes their maturation. The MH is then used for reproductive regulation and induces the synthesis of vitellogenin (Vg) in fat bodies [12,13]. In the early stages of female oogenesis, disruption of the MH signaling pathway can cause changes in ovarian stem cells and may interfere with meiosis and the development of primary oocytes [14,15]. In the later stages of oogenesis, the MH can regulate the synthesis of yolk proteins by fat bodies, the absorption of yolk granules by oocytes, egg maturation, and egg deposition [16,17]. The MH can also impact the development of male accessory glands and sperm formation by regulating mitosis and meiosis [18]. When 20E was injected into silkworms or added to mulberry leaves, *BmVg* transcription was induced in *Bombyx mori* fat bodies [19]. In *Tribolium castaneum*, RNA interference (RNAi) demonstrated that *EcR* and *USP* were essential for ovary development, oocyte maturation, and the growth and migration of follicle cells [16].

Previous studies have shown that adding the MH or juvenile hormones (JHs) to artificial diets caused a significant increase in the reproductive capacity of ladybugs [20]. RNAi studies showed that genes encoding the methotrexate receptor (*Met*) and the transcription factor krüppel homolog 1 (*Kr-h1*) on the JH signaling pathway play important roles in regulating the reproduction of ladybugs by directly affecting Vg formation, ovary and testis development, and fertility [21,22]. Previous studies focused on the role of the MH signaling pathway on larval molting, whereas research on the reproductive function of *EcR* in adults was limited to a relatively few insect species. Consequently, relevant studies on the reproductive regulation of ladybugs have been inadequate. In this report, we use RNAi to investigate the regulatory function of *EcR* in *C. septempunctata* reproduction. The results contribute to our knowledge of reproductive regulation in ladybugs and provide further guidance in formulating artificial diets for ladybugs.

## 2. Materials and Methods

### 2.1. Insects

The ladybugs used herein were obtained from a population raised in a growth chamber at the Guizhou Institute of Plant Protection. This population was reared on aphids [*Aphis craccivora* Koch (Hemiptera: Aphididae)] as a food source and maintained in growth chambers at 70 ± 5% RH, 25 ± 1 °C, and under a 16:8 h light/dark photoperiod [23].

### 2.2. Ladybug Diets

The compounds used in artificial diets were obtained and formulated as reported previously [20]. The methods for rearing ladybugs on *A. craccivora* and aphids on horsebean seedlings have been described [23].

The compounds in diet 1 were described in a prior publication [22] and included the following: milk powder, 15 g; pig liver, 105 g; eggs, 10 g; olive oil, 2 g; corn oil, 2 g; casein, 7.5 g; cholesterol, 5 g; sucrose, 45 g; protein powder, 4.5 g; powdered yeast, 0.5 g; vitamin C, 1 g; honey, 7.5 g; vitamin E, 1 g; sterile water, 370 g; and agar, 6.17 g.

The diet 2 included all substances in diet 1 as well as 1.7 mg of 20-hydroxyecdysone (95.0%, Shanghai Acmec Biochemical Co., Shanghai, China).

### 2.3. Sample Handling and Collection

Selected 1 d old ladybug adults were fed on aphids or diet 1 or diet 2. Five- and ten-day-old ladybugs were reared on diet 1 or 2 or aphids. Each sample contained four adults, and each treatment was repeated three times. The collected samples were transferred to 10 mL centrifuge tubes washed in 1 × PBS buffer, dried, frozen in liquid nitrogen, and stored at −80 °C until needed.

### 2.4. RNA Extraction and cDNA Synthesis

Ladybug adults were ground to a powder in liquid nitrogen, and RNA was obtained using the Eastep^®^ Super RNA kit (Promega, Beijing, China) as described [22]. The iScript cDNA Synthesis Kit (Bio-Rad, Hercules, CA, USA) was utilized to synthesize cDNA templates using established protocols [22]. Samples were stored at −20 °C until needed.

### 2.5. RNA Interference

#### 2.5.1. Synthesis of EcR dsRNA

Based on previous transcriptome sequencing of *C. septempunctata* by our research group [24], the *EcR* and *E78* sequences were obtained, and primers were designed to synthesize double-stranded RNA (*EcR*-dsRNA) (Table 1). T7 promoter sequences were incorporated into the 5′ end of *EcR*-dsRNA primers. Ladybug DNA served as the template, and DNA fragments specific for *EcR* and *GFP* (encoding green fluorescent protein) were amplified using the primers in Table 1 as described [21]. The established protocols and primers EcR-dsRNA-F/R and GFP-dsRNA-F/R (Table 1) were used to synthesize *EcR*-dsRNA and *GFP*-dsRNA, respectively [22].

#### 2.5.2. Injection of dsRNA

Newly emerged one-day-old adults were injected with 1 μL *EcR*-dsRNA or *GFP*-dsRNA (sham-injected controls) using the Eppendorf Transferman 4R Micromanipulator (Eppendorf, Germany). The adult ladybugs were placed in 25 mL plastic bottles and exposed to CO_2_ for 15 s; the CO_2_-exposed adults were then removed, viewed with a stereomicroscope, and injected at the 3rd and 4th abdominal intermembrane. The needle was withdrawn 5 s after injection to prevent excess liquid from flowing out of the injected ladybugs. Each treatment consisted of 50 adults, and experiments were repeated three times.

#### 2.5.3. Effects of RNAi on Ladybug Adults

Three RNAi experiments were conducted. In the first experiment, adult ladybugs were injected with dsRNA and supplied with an aphid diet. Adults were selected on the 5th and 10th d after injection; RNA was extracted, cDNA was synthesized, and expression was analyzed by quantitative real-time PCR (qPCR). Each sample contained four adults, and treatments contained three biological replicates. In the second experiment, adults were injected with dsRNA as described above, and the ovaries and testes were removed by dissection at 5 and 10 d post-injection. Reproductive organs were examined with a stereomicroscope and measured with Image View software (x64, 4.11.18709.20210403). The following measurements were taken: ovary length, lengths and widths of the left and right egg chambers, accessory gland lengths and widths, and testis lengths. Thirty dissected adults were used from each treatment. The ovary and testes were quantitatively detected using the Bradford method for protein.

In the third experiment, egg production was measured in two groups of mating pairs. Group I consisted of females injected with dsRNA and noninjected males, whereas group II comprised males injected with dsRNA and noninjected females. Each treatment consisted of three replicates, with 10 pairs of ladybugs per replicate. Egg production was recorded for 20 days after injection.

### 2.6. Quantitative Real-Time PCR (qPCR)

The EcR-F/EcR-R primers (Table 1) were used to measure *EcR* expression in ladybugs. qPCR was performed in 10 μL containing the cDNA template (1 μL), EcR-F/EcR-R primers (1 μL each), 5 μL SsoAdvanced Universal SYBR Green Supermix (Bio-Rad, Hercules, CA, USA), and 2 μL ddH_2_O. The reaction conditions for qPCR included 2 min at 95 °C (pre-denaturation), 5 s at 95 °C (denaturation), and 30 s of 60 °C for 39 cycles (annealing and extension). *Actin* expression was determined with Actin-F/R primers (Table 1) following annealing temperatures described previously [25]. Melting curve analysis was conducted to determine primer specificity after the reaction. All experiments were conducted using three biological and three technical replicates. The 2^−∆∆Ct^ method was used to obtain relative expression levels [26].

### 2.7. Data Analysis

Data were analyzed with DPS v.19.05 [27]. Two treatments were compared using the *t*-test, and three treatments were analyzed using one-way analysis of variance (ANOVA). Duncan’s multiple comparison method was used to assess significance at *p* < 0.05, and the data points shown represent means ± standard error (SE).

## 3. Results

### 3.1. 20-Hydroxyecdysone-Mediated Changes in EcR Transcription

**Figure 1:**
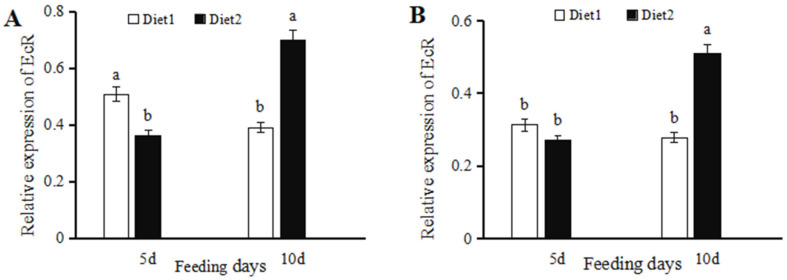
Relative expression of *EcR* in *Coccinella septempunctata* at five and ten days after feeding on diet 1 or 2. Diet 2 is diet 1 amended with 20-hydroxyecdysone. Panels show the expression of *EcR* in females (**A**) and males (**B**). Column heights represent means ± SE (*n* = 3), and columns labeled with different letters indicate a significant difference at *p* < 0.05 using the *t*-test.

*EcR* expression in female adults fed with diet 2 for five days was significantly reduced (28.45% lower) (t = 3.3211, *p* = 0.0293) as compared to those supplied with diet 1 (Figure 1A). In contrast, *EcR* expression levels in females fed with diet 2 for ten days were significantly higher (78.41%) (t = 3.6314, *p* = 0.0221) than females fed with diet 1. *EcR* expression in male adults supplied with diet 2 for five days was 13.32% lower than those on diet 1, and the difference was not significant (t = 0.6717, *p* = 0.5386) (Figure 1B). However, *EcR* expression in males supplied with diet 2 for ten days was significantly higher (83.24%) (t = 7.7019, *p* = 0.0015) than the expression in those fed on diet 1.

### 3.2. Effects of dsRNA on EcR Expression

**Figure 2:**
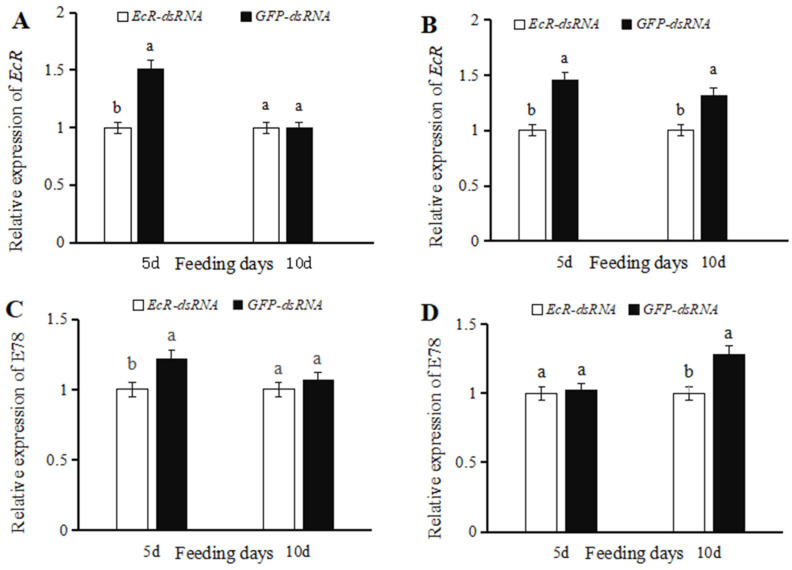
Relative expression of *EcR* and *E78* in *Coccinella septempunctata* at five and ten days after injection with *EcR*-dsRNA or *GFP*-dsRNA. Panels show the relative expression of *EcR* in females (**A**) and males (**B**). Panels show the relative expression of *E78* in females (**C**) and males (**D**). Column heights indicate means ± SE (*n* = 3), and the labeling of columns with different letters indicates significance at *p* < 0.05 using the *t*-test.

*EcR* expression levels in female ladybugs injected with *EcR*-dsRNA were significantly reduced (33.96% lower) (t = 3.8289, *p* = 0.0186) than insects microinjected with *GFP*-dsRNA at five days (Figure 2A). No significant differences in *EcR* expression were observed in females injected with *EcR*-dsRNA or *GFP*-dsRNA at ten days post-injection (t = 0.0467, *p* = 0.9650) (Figure 2A). *EcR* expression levels in males treated with *EcR*-dsRNA and analyzed at five days and ten days were significantly reduced (31.22% and 24.19% lower, respectively) (t = 5.0927, *p* = 0.0070) as compared to males treated with *GFP*-dsRNA (Figure 2B).

*E78* expression levels in female ladybugs injected with *EcR*-dsRNA were significantly reduced (17.97% lower) (t = 2.8759, *p* = 0.0452) than insects microinjected with *GFP*-dsRNA at five days (Figure 2C). No significant differences in *E78* expression were observed in females injected with *EcR*-dsRNA or *GFP*-dsRNA at ten days post-injection (t = 0.4474, *p* = 0.6984) (Figure 2C). *E78* expression levels in males treated with *EcR*-dsRNA at five days showed no significant differences (t = 0.1301, *p* = 0.9028) as compared to males treated with *GFP*-dsRNA (Figure 2D). *E78* expression levels in males injected with *EcR*-dsRNA were significantly reduced (21.98% lower) (t = 3.0271, *p* = 0.0389) than insects microinjected with *GFP*-dsRNA at ten days (Figure 2D).

### 3.3. Effects of dsRNA Injection on Ovaries and Testes

**Figure 3:**
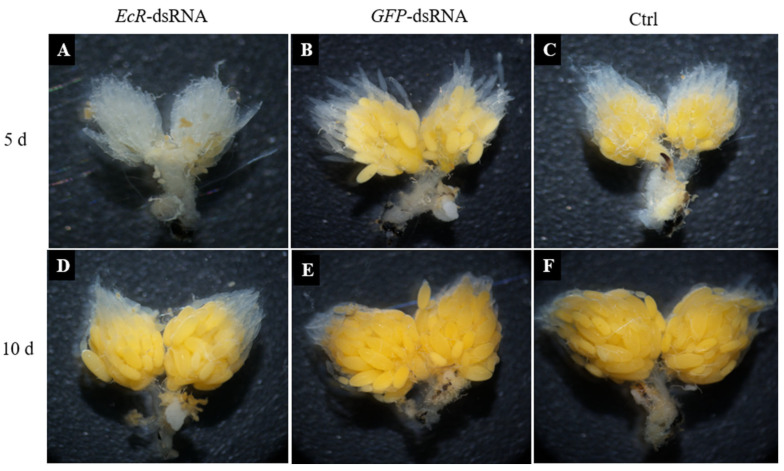
Ovary development in *Coccinella septempunctata* females after injection with *EcR*-dsRNA or *GFP*-dsRNA. Panels (**A**,**B**) show ovary development in females at 5 d after injection with *EcR*-dsRNA and *GFP*-dsRNA, respectively. Panels (**D**,**E**) show ovary development in females at 10 d after injection with *EcR*-dsRNA and *GFP*-dsRNA, respectively. Control (Ctrl) panels (**C**,**F**) show ovary development in noninjected females.

Ovary formation in female adults was evaluated at five days and ten days after microinjection with *EcR*-dsRNA and *GFP*-dsRNA. Very few eggs were produced in the ovaries of ladybugs injected with *EcR*-dsRNA at five days, whereas eggs in *GFP*-dsRNA-injected females filled approximately half of the ovary at five days (Figure 3A,B). When sampled ten days after injection with *EcR*-dsRNA, approximately two-thirds of the ovaries were filled with eggs, whereas eggs in females microinjected with *GFP*-dsRNA filled the entire ovary (Figure 3D,E). No obvious differences were noted in the egg development of females treated with *GFP*-dsRNA and the noninjected control.

**Figure 4:**
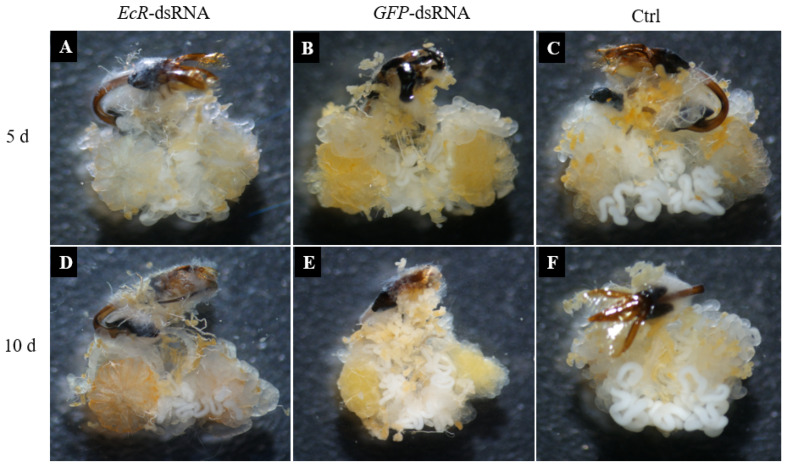
Testis development in *Coccinella septempunctata* males after microinjection with *EcR*-dsRNA or *GFP*-dsRNA. Panels (**A**,**B**) illustrate testes in males at five days following injection with *EcR*-dsRNA and *GFP*-dsRNA, respectively. Panels (**D**,**E**) show testis development in males at ten days following injection with *EcR*-dsRNA and *GFP*-dsRNA, respectively. Control (Ctrl) panels (**C**,**F**) show the development of testes in males that were not injected.

Testis development in male adults was evaluated at five days and ten days post-injection with *EcR*-dsRNA or *GFP*-dsRNA. The formation of testes in males injected with *EcR*-dsRNA was delayed at both time points as compared to males treated with *GFP*-dsRNA (Figure 4A,D). Males treated with *GFP*-dsRNA had more white substances (containing semen proteins [28]) in the accessory glands, whereas the group injected with *EcR*-dsRNA had fewer white substances and contained collapsed testicular tubes. No obvious differences were apparent in the formation of white substances in the accessory glands of *GFP*-dsRNA-injected and noninjected males.

**Figure 5:**
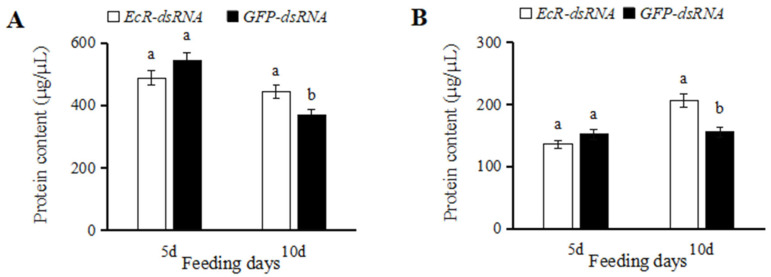
Protein concentration in *Coccinella septempunctata* after microinjection with *EcR*-dsRNA or *GFP*-dsRNA. Panels show protein concentration in females (**A**) and males (**B**). Column heights represent means ± SE (*n* = 3), and columns labeled with different letters indicate a significant difference at *p* < 0.05 using the *t*-test.

After injecting *EcR*-dsRNA into *C. septempunctata* for five days, the protein content of female and male insects was lower than that of *GFP*-dsRNA-injected insects, and the difference was not significant (Figure 5A,B). However, at 10 d after injection, the protein content in insects treated with *EcR*-dsRNA was significantly higher than that of *GFP*-dsRNA-treated insects (Figure 5A,B).

### 3.4. Effects of dsRNA Injection on Development of Reproductive Systems

**Figure 6:**
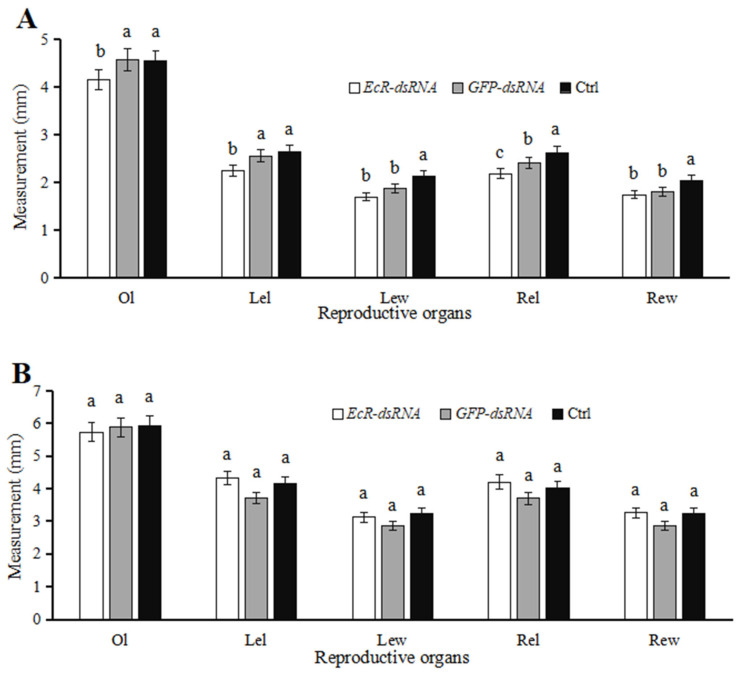
Ovary measurements in female ladybugs after microinjection with *EcR*-dsRNA or *GFP*-dsRNA. Columns labeled as Ctrl represent noninjected controls. Panels show ovary measurements at five (**A**) and ten (**B**) days post-injection. Abbreviations: Ol, ovary length; Lel, length of left egg chamber; Lew, width of left egg chamber; Rel, length of right egg chamber; and Rew, width of right egg chamber. Data points represent means ± SE (*n* = 3). Columns labeled with different letters indicate significance at *p* < 0.05 using Duncan’s test.

The ovary length (Ol), width of the left egg chamber (Lew), length of the left egg chamber (Lel), width of the right egg chamber (Rew), and length of the right egg chamber (Rel) in ladybugs injected with *EcR*-dsRNA decreased by 9.14%, 9.28% 12.05%, 3.16%, and 8.93% at five days, respectively, as compared to ladybugs microinjected with *GFP*-dsRNA (Figure 6A). Among these, the lengths of the ovaries and the right and left egg chambers were significantly lower (*p* < 0.05) for *EcR*-dsRNA-injected females than in *GFP*-dsRNA-treated females (Figure 6A). Interestingly, ladybugs injected with *EcR*-dsRNA, analyzed at ten days, showed increases of 16.48%, 9.43%, 13.48%, and 13.93% in Lel, Lew, Rel, and Rew, respectively, when compared to the *GFP*-dsRNA group (Figure 6B); however, these differences were not significant.

**Figure 7:**
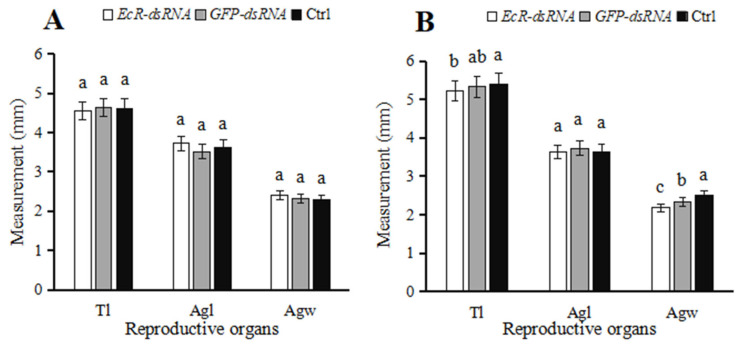
Testis measurements in *Coccinella septempunctata* after microinjection with *EcR*-dsRNA or GFP-dsRNA. Columns labeled as Ctrl represent noninjected controls. Panels: testis measurements at (**A**) five days and (**B**) ten days after microinjection. Abbreviations: Tl, length of testes; Agl, length of accessory glands; Agw, width of accessory glands. Data points represent means ± SE (*n* = 3). Columns with different letters represent significance at *p* < 0.05 using Duncan’s test.

In ladybugs injected with *EcR*-dsRNA, testis lengths decreased by 2.13% and accessory gland lengths and widths increased by 5.79% and 3.47%, respectively, as compared to insects microinjected with *GFP*-dsRNA at five days (Figure 7A); however, these differences were not significant at *p* > 0.05. At ten days after microinjection with *EcR*-dsRNA, testis lengths, accessory gland lengths, and accessory gland widths decreased by 1.74%, 2.54%, and 7.03%, respectively, when compared to males microinjected with *GFP*-dsRNA (Figure 7B). In the *GFP*-dsRNA-treated group, there were no significant differences in testis lengths and accessory gland lengths at 10 d when compared to the noninjected group (*p* > 0.05), indicating that the injection wound did not have a significant impact on the male testes.

### 3.5. Effect of dsRNA Injection on Egg Production and Hatching

**Figure 8:**
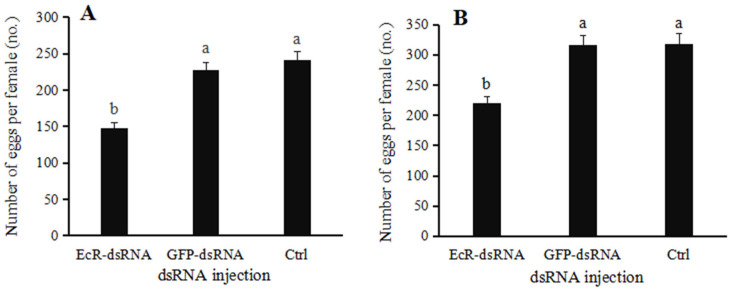
Fecundity of *Coccinella septempunctata* following microinjection with *EcR*-dsRNA or *GFP*-dsRNA. (**A**) Fecundity of females after injection with *EcR*-dsRNA or *GFP*-dsRNA and mating with males. (**B**) Fecundity of males after injection with *EcR*-dsRNA or *GFP*-dsRNA and mating with females. Ctrl, noninjected control. Data points represent mean ± SE (*n* = 3). Columns with different letters indicate significance at *p* < 0.05 using Duncan’s test.

**Figure 9:**
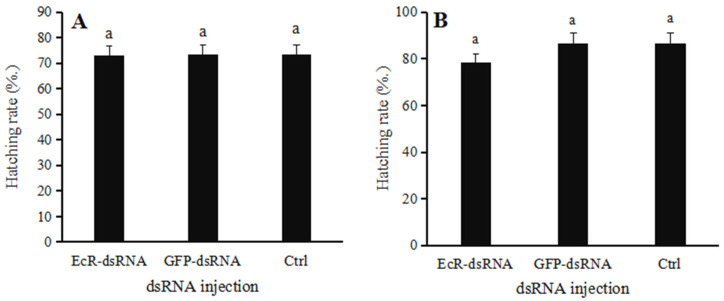
Hatching rates of *Coccinella septempunctata* following microinjection with *EcR*-dsRNA or *GFP*-dsRNA. (**A**) Hatching rate of females after injection with *EcR*-dsRNA or *GFP*-dsRNA and mating with males. (**B**) Hatching rate of males after injection with *EcR*-dsRNA or *GFP*-dsRNA and mating with females. Ctrl, noninjected control. Data points represent mean ± SE (*n* = 3). Columns with different letters indicate significance at *p* < 0.05 using Duncan’s test.

When female ladybugs were treated with *EcR*-dsRNA or *GFP*-dsRNA, paired with noninjected males and reared for twenty days, the mean egg production was 148 and 227 eggs, respectively (Figure 8A), whereas the mean egg production in the noninjected group was 241 eggs. In females injected with *EcR*-dsRNA, egg production was significantly lower than that of the *GFP*-dsRNA group (F = 82.5768, *p* = 0.0006). The hatching rates of female insects injected with *EcR*-dsRNA decreased when paired with males, but the difference was not significant when compared to *GFP*-dsRNA-injected or noninjected females (F = 0.0376, *p* = 0.9634) (Figure 9A).

When males were treated with *EcR*-dsRNA or *GFP*-dsRNA and paired with noninjected females, the mean egg production for twenty days was 220 and 316 eggs, respectively (Figure 8B). Interestingly, when males were microinjected with *EcR*-dsRNA, egg production by females was significantly reduced (F = 26.5189, *p* = 0.0049); however, microinjection of males with *GFP*-dsRNA had no significant impact on fecundity, similar to the noninjected control group. The hatching rates of male insects injected with *EcR*-dsRNA decreased when paired with females, but the difference was not significant when compared to *GFP*-dsRNA-injected or noninjected males (F = 2.9089, *p* = 0.1600) (Figure 9B).

## 4. Discussion

The role of ecdysteroids in adult insects is mediated by receptors, especially the heterodimeric nuclear receptor EcR/USP. In this study, ladybug adults supplied with an artificial diet containing 20E showed decreased *EcR* expression at five days; this may be the outcome of increased 20E metabolism and concomitant binding to *EcR* receptors, resulting in decreased *EcR* expression. Furthermore, *EcR* expression was upregulated up to ten days when ladybug adults were supplied with an artificial diet supplemented with 20E. We speculated that the addition of 20E to artificial diets may increase *EcR* transcription with prolonged feeding, as the amount of *EcR* consumed in 20E metabolism was less than the synthesis amount. When 20E was supplied to *Antheraea pernyi* pupae, diapause was induced, and *ApEcRB1* and *ApUSP1* expression decreased [11]. *ApEcRB1* expression remained low for 12 d in the later stages of pupal development; however, expression sharply increased in the early stages of eclosion and reached its highest level at 16 d of pupal development. Similarly, *ApUSP1* expression levels began to increase at 8 d after injection and reached peak levels at 16 d [11]. In *Apis mellifera*, expression levels of *EcRA*, *EcRB1*, and *USP1* began to decrease 1 h after injection with 20E, and high concentrations of 20E inhibited *EcR* expression [29]. In a subsequent study, Yu et al. [30] attempted to rescue *EcRA* and *USP* expression in *A. mellifera EcR*-RNAi larvae by injecting 20E, but the expression levels remained low.

The MH receptor *EcR* is positioned at the onset of cascading reactions that control insect molting, metamorphosis, and reproduction, and *EcR* plays an essential role in these processes. The 20E signaling mediated by *EcR* and *USP* can control the differentiation of germline stem cells during early development of the ovaries [31]. In *Drosophila* female adults, oogenesis in *EcR* mutants is defective, and the spectrum of oogenic defects includes the presence of abnormal egg chambers and loss of vitellogenic egg stages [32]. *EcR* and *USP* were significantly inhibited at the transcriptional level 6–18 days after injection of dsEcR and dsUFP, and the number of eggs in the ovaries and eggs laid per female significantly decreased compared with the control [33]. In *Spodoptera littoralis* male adults, injection of 20E inhibited and delayed the release of sperm bundles [34]. The downregulation of molting-hormone-responsive genes (e.g., *E75*, *E78*, *FTZ-F1*, *HR4*, *HR38*, *HR39*) in male *T. castanenum* can lead to abnormal accessory gland development, reduced sperm count, and a significant decrease in egg production in mating females. However, *EcR*-RNAi and *USP*-RNAi had no significant effect on male reproduction in *T. castanenum*, which may be due to insufficient interference or the presence of other functional ecdysteroid receptors [35]. In our study, injection of *EcR*-dsRNA into female ladybugs inhibited *EcR* and *E78* transcription, delayed ovary development, and reduced egg production. After *EcR*-dsRNA was microinjected into male adults, testis volumes decreased and substances in the accessory gland (primarily semen proteins) decreased. Furthermore, egg production decreased when males injected with *EcR*-dsRNA were paired with noninjected females. These results indicate that the reproductive abilities of both male and female ladybug adults are regulated by *EcR*.

*EcR* is widely present in insect tissues, including the central nervous system, fat body, intestines, and reproductive organs of both male and female insects [10]. Fat bodies are responsible for providing yolk protein precursors and the energy required for egg maturation [36,37]. Kamoshida et al. [38] found that *EcR*-mediated ecdysteroid signaling can reduce lipid accumulation in *Drosophila* fat bodies. Further research is needed to evaluate whether lipid accumulation in the fat body of *C. septempunctata* is regulated by *EcR*.

## 5. Conclusions

The results clearly demonstrate that *EcR* is required for normal ovary and testis development and maximal egg production in *C. septempunctata*, indicating that *EcR* has a bifunctional role in the reproduction of males and females. This study further contributes to the improvement of methods for cultivating natural enemies and will ultimately benefit pest control.

## Figures and Tables

**Table 1:** Sequences of primers used in this study.

Primer Name	Sequence	Annealing Temperature
EcR-F EcR-R	AAAGGACCAACACCAAGGCA TCGTCACACTCCGTCGAAAG	55.5
EcR-dsRNA-F EcR-dsRNA-R	taatacgactcactatagggCGATGACTTGCTGCTGGTTA taatacgactcactatagggGTCGTAGCTGCCTGATGACA	
GFP-dsRNA-F GFP-dsRNA-R E78-F E78-R	taatacgactcactatagggGCCAACACTTGTCACTACTT taatacgactcactatagggGGAGTATTTTGTTGATAATGGTCTG AGCTCCTGTTTCATGATGCGG ATTCATCCCCGGTCGAATGTG	58.0
Actin-F Actin-R	GATTCGCCATCCAGGACATCTC TCCTTGCTCAGCTTGTTGTAGTC	60.0

Letters in lowercase indicate the T7 promoter region.

## Data Availability

The original contributions presented in this study are included in the article. Further inquiries can be directed to the corresponding author.

## References

[1] Cartwright B.O., Eikenbary R.D., Angalet G.W., Ampbell R.K. (1979). Release and establishment of *Coccinella septempunctata* in Oklahoma. Environ. Entomol..

[2] Hance T., Dixon A.F.G. (2006). Insect predator-prey dynamics: Ladybird beetles and biological control. J. Insect Conserv..

[3] Hodek I., Michaud J.P. (2008). Why is *Coccinella septempunctata* so successful? (A point-of-view). Eur. J. Entomol..

[4] Duan Y.J., He H.G., Pu D.Q., Yang H., Chen Q., Lu Q.C. (2021). Basic research status of *Coccinella septempunctata*. J. Biosaf..

[5] Smirnoff W.A. (1958). An artificial diet for rearing Coccinellid beetles. Can. Entomol..

[6] Yang D.L., Hao J., Wang Y.N., Xi G.S. (2014). Research progress of ecdysone in adult insects. Chin. Bull. Life Sci..

[7] Niwa R., Niwa Y.S. (2014). Enzymes for ecdysteroid biosynthesis: Their biological functions in insects and beyond. Biosci. Biotech. Bioch..

[8] Zhu J.S. (2022). Non-genomic action of juvenile hormone modulates the synthesis of 20- hydroxyecdysone in *Drosophila*. Sci. Bull..

[9] Feyereisen R. (2012). Insect CYP genes and P450 enzymes. Insect Mol. Miol..

[10] Wang J.J., Hu Q.B. (2017). A review of ecdysone receptor. J. Environ. Entomol..

[11] Ru Y.T., Wang Y., Zhou J.G., Wang D.Y., Ma Y.Y., Jiang Y.R., Gao Q., Qin L. (2017). The expression patterns of ecdysone receptor and ultraspiracle genes in *Antheraea pernyi* during development and hormone-induced process. Sci. Seric..

[12] Fallon A.M., Hagedorn H.H., Wyatt G.R., Laufer H. (1974). Activation of vitellogenin synthesis in the mosquito *Aedes aegypti* by ecdysone. J. Insect Physiol..

[13] Zhou J., Li J., Wang Q., Luo Y.Q. (2013). The regulation of ecdysteroid on insect growth and productive processes. Chinese J. Applied Entomol..

[14] König A., Yatsenko A.S., Weiss M., Shcherbata H. (2011). Ecdysteroids affect *Drosophila* ovarian stem cell niche formation and early germline differentiation. EMBO J..

[15] Morris L.X., Spradling A.C. (2012). Steroid signaling within *Drosophila* ovarian epithelial cells sex-specifically modulates early germ cell development and meiotic entry. PLoS ONE.

[16] Parthasarathy R., Sheng Z.T., Sun Z.Y., Palli S.R. (2010). Ecdysteroid regulation of ovarian growth and oocyte maturation in the red flour beetle, *Tribolium castaneum*. Insect Biochem. Molec..

[17] Swevers L., Iatrou K. (2009). Ecdysteroids and ecdysteroid signaling pathways during insect oogenesis. Ecdysone: Structures and Functions.

[18] Happ G.M. (1992). Maturation of the male reproductive system and its endocrine regulation. Annu. Rev. Entomol..

[19] Shen G.W., Lin Y., Lv Y.H., Wang J.Y., Xing R.M., Xia Q.Y. (2014). Regulation of ecdysone on vitellogenin gene expression in silkworm *Bombyx mori*. Chin. J. Biochem. Mol. Biol..

[20] Cheng Y., Zhou Y.H., Ran H.Y., Li F.L. (2023). Effects of different hormones as dietary supplements on biological characteristics of *Coccinella septempunctata* L. J. Appl. Entomol..

[21] Cheng Y., Zhou Y., Li C., Jin J.X. (2024). Cloning and functional analysis of the juvenile hormone receptor gene *CsMet* in *Coccinella septempunctata*. J. Insect Sci..

[22] Cheng Y., Zhou Y., Li C. (2025). Functional analysis of genes encoding juvenile hormone receptor *Met* and transcription factor *Kr-h1* in the reproductive capacity of *Coccinella septempunctata* males. Insects.

[23] Zhou Y.H., Cheng Y., Jin J.X., Li W.H., Li F.L. (2017). Large scale production and release application of *Coccinella septempunctata*. Southwest China J. Agric. Sci..

[24] Cheng Y., Zhi J.R., Li F.L., Wang H., Zhou Y.H., Jin J.X. (2020). Transcriptome sequencing of *Coccinella septempunctata* adult (Coleoptera: Coccinellidae) feeding on artificial diet and *Aphis craccivora*. PLoS ONE.

[25] Liu M.Y., Wang J., Wang M.Z., Gao F., Zhang H.Z., Li Y.Y., Zang L.S., Zhang L.S. (2019). Cloning and expression analysis of juvenile hormone epoxide hydrolase in *Coccinella septempunctata*. Plant Prot..

[26] Pfaffl M.W. (2001). A new mathematical model for relative quantification in real-time RT-PCR. Nucleic. Acids Res..

[27] Tang Q.Y., Zhang C.X. (2013). Data Processing System (DPS) software with experimental design, statistical analysis and data mining developed for use in entomological research. Insect Sci..

[28] Wang Z.S., Zhong X.C., Qiu X.J., Hu S.Y., Guo F. (1977). Observations on the reproduction of *Coccinella septempunctata* L. Acta Entomol. Sin..

[29] Mello T.R.P., Aleixo A.C., Pinheiro D.G., Nunes F.M.F., Bitondi M.M.G., Hartfelder K., Barchuk A.R., Simoes Z.L.P. (2014). Developmental regulation of ecdysone receptor (EcR) and EcR-controlled gene expression during pharate-adult development of honeybees (*Apis mellifera*). Front. Genet..

[30] Yu J., Song H.Y., Wang Y., Liu Z.G., Wang H.F., Xu B.H. (2023). 20-hydroxyecdysone upregulates ecdysone receptor (EcR) gene to promote pupation in the honeybee, *Apis Mellifera* Ligustica. Integr. Comp. Biol..

[31] Ables E.T., Drummond-Barbosa D. (2010). The steroid hormone ecdysone functions with intrinsic chromatin remodeling factors to control female germ line stem cells in *Drosophila*. Cell Stem Cell.

[32] Carney G.E., Bender M. (2000). The *Drosophila* ecdysone receptor (EcR) gene is required matemally for normal oogenesis. Genetics.

[33] Huang X.X., Zheng W.Y., Xue H., Jiao P.Y., Chen L.Z. (2023). Effects of silencing ecdysone receptor gene on the fecundity of *Adelphocris suturalis*. Plant Prot..

[34] Polanska M.A., Maksimiuk-Ramirez E., Ciuk M.A., Kotwica J., Bebas P. (2009). Clock-controlled rhythm of ecdysteroid levels in the haemolymph and testes and its relation to sperm release in the egyptian cotton leafworm, *Spodoptera littoralia*. J. Insect Physiol..

[35] Xu J., Raman C., Zhu F., Tan A., Palli S.R. (2012). Identification of nuclear receptors involved in regulation of male reproduction in the red flour beetle, *Tribolium castaneum*. J. Insect Physiol..

[36] Attardo G.M., Hanseni A., Raikhel A.S. (2005). Nutritional regulation of vitellogenesis in mosquitoes: Implications for anautogeny. Insect Biochem. Molec..

[37] Wang X.L., Hou Y., Saha T.T., Pei G.F., Raikhel A.S., Zou Z. (2017). Hormone and receptor interplay in the regulation of mosquito lipid metabolism. Proc. Nati. Acad. Sci. USA.

[38] Kamoshida Y., Fujiyama-Nakamur S., Kimura S., Suzuki E., Lim J., Shiozaki-Sato Y., Kato S., Takeyama K. (2012). Ecdysone receptor (EcR) suppresses lipid accumulation in the *Drosophila* fat body via transcription control. Biochem. Biophys. Res. Commun..

